# Health Status and Association With Interpersonal Relationships Among Chinese Children From Urban Migrant to Rural Left-Behind

**DOI:** 10.3389/fpubh.2022.862219

**Published:** 2022-03-29

**Authors:** Guanlan Zhao, Menmen Wang, Jiayao Xu, Jingjing Lu, Hailati Akezhuoli, Feng Wang

**Affiliations:** ^1^Institute of Social Medicine, Zhejiang University School of Medicine, Hangzhou, China; ^2^School of Public Health, Hangzhou Normal University, Hangzhou, China

**Keywords:** migrant children, left-behind children, interpersonal relationship, health status, China

## Abstract

**Background:**

To date, numerous studies have examined the health status of Chinese left-behind children and migrant children. However, the impact of children's diverse migration/left-behind experiences on their health is still unclear.

**Methods:**

A cross-sectional survey was conducted in 2020 in Nanling country (Anhui province) and Kaihua country (Zhejiang province) in China. School children from grade 5 to 8 reported their socio-demographic, interpersonal relationships, self-rated health, suicidal ideation, and depression. Participants were divided into four groups based on their migrant patterns, namely rural left-behind children with previous migration experience (ME-LBC), rural children with previous migration experience (ME-NLBC), rural left-behind children without migration experience (LBC), and rural children without migration experience (NLBC).

**Results:**

Among 2,323 participants included in the present study, there were 336 ME-LBC (14.5%), 283 ME-NLBC (12.2%), 561 LBC (24.1%) and 1,143 NLBC (49.2%). Compared with NLBC, ME-LBC reported significantly poorer self-rated health (OR = 0.72, 95% CI [0.53–0.97], *p* < 0.05), higher risk of depression (β = 0.90, 95% CI [0.02–1.77], *p* < 0.05) with adjustment of socio-demographic and interpersonal relationships. There was no significant difference in suicidal ideation among different groups of children. The better interpersonal relationship was associated with a better self-rated health, and lower prevalence of depression and suicidal intention.

**Conclusions:**

Compared to ordinary rural children, ME-LBC tended to experience higher levels of depression and poorer self-rated health. These research findings imply developing intervention programs about psychological adjustment tailored to different migrant patterns of Chinese rural children. The keys might be to strengthen the relationships with peer and teacher in school and improve the quality of parent-child communication in family for LBC.

## Introduction

With the rapid social and economic development, there has been continued growth of rural-to-urban migration in China over the past 30 years (1). This phenomenon has been considered as the largest migration in human history (2), where more than 172 million rural people have migrated from their hometowns to cities for better employment opportunities (3). As a result, a great number of Chinese rural children have experienced parental migration during their childhood. Children of these migrant parents can benefit from their migrant parents' increased income and subsequently improved family economic status. It is a dilemma for those children to decide to move with their migrant parents or to stay. Moving can put those children under an unfamiliar and unstable environment, while staying can leave those children living in a circumstance lacking parental supervision and support due to parental absence. In either case, children's mental health and future development can be negatively affected.

### Mental Health of Migrant and Left-Behind Children

There is an increasing number of migrant parents choosing to raise their children in cities, resulting in the “migrant children” (MC) (4). Migrant children are migrant population under the age of 18 years who have left their hukou registration place for 6 months or longer with parents from rural areas to cities (5). There are 34.26 million MC, accounting for approximately 12.8% of Chinese children (6). There is also a tremendous number of migrant parents who can't afford to raise their children in cities but to leave their children in the rural hometown, resulting in the “left-behind children” (LBC). Left-behind children are defined as population under the age of 18 years who stay at their hukou registration place while both of their parents or one parent migrate to urban areas to work for at least 6 months (7). There are approximately 41 million rural LBC, accounting for 29% of rural children and 15% of Chinese children (8). LBC are usually taken care of by their grandparents or their families.

The mental health of LBC in rural areas and MC in urban areas have drawn great attention from researchers across many disciplines (e.g., public health, sociology, psychology, anthropology). A number of previous studies has showed the mental health outcomes in LBC and MC, although the results were inconsistent, most findings have documented the adverse effects on child's mental health due to the long-term lack of parental care. One meta-analysis which included 91 Chinese studies demonstrated that LBC were 52% more likely to report depression, 70% more likely to report suicidal ideation, and 85% more likely to report anxiety due to parental absence and the subsequent insufficient parent-child communication compared with rural children (9). Previous studies also showed that compared with urban children, MC experienced more mental health problems such as depression and loneliness (10, 11), which can be explained by stressful events such as peer rejection, academic challenges, family conflicts and social isolation due to their migrant experiences (12, 13).

### Diverse Migrant Experience and Child Mental Health

Migration is a fluid process. Different from attributes such as gender and ethnicity, being “left-behind” or “migrant” is a temporary status of some children in their growth process, rather than a specific attribute accompanying them throughout their lives. Some children even have mixed experiences of being left-behind and being migrants at different times. Literature has focused on the fluidity and heterogeneity of children's migrant experiences (3). Because of the *Hukou*, the Chinese Household Registration System determining the distribution of social welfare resources including employment opportunities, medical care, and education resources (14, 15), many school-aged MC are limited to access public schools or social welfare. Thus, many MC may return to their hometowns by school-age and become *returning migrant children* (16). Quite a few rural children even have mixed experiences of being left-behind and being migrant at different times. According to a rough estimation by the All-China Women's Federation, there were nearly 3.5 million migrant children who returned to their hometowns in 2009 (16).

Migrant children returning to their hometowns without the presence of both parents were classified as rural left-behind children with previous migration experience (ME-LBC) in the present study. ME-LBC are different from LBC because they necessitate a re-adaptation to a no-longer-familiar educational and social environment in rural areas (16). They are also different from MC because they move from urban areas back to rural areas and have to rebuild new interpersonal relationships without the presence of both parents and may also have to build attachment with their new caregivers if they move back alone. Koo et al. described this group as “a doubly disadvantaged child population” because they suffered from damaged social ties with interpersonal relationships in the school and the larger community due to residential relocation as well as separation from parents if they returned alone (17). To our knowledge, few researchers have measured the mental health of ME-LBC. Liu and Zhu's qualitative study reported that over a third of returning migrant children experienced difficulties in re-adapting to rural life (16). Previous migration experiences significantly contributed to the increased risks of depression and impairments of self-esteem and resilience of rural Chinese children (18). So far, literature on this topic remains relatively limited.

### Child Mental Health and Interpersonal Relationships

The close interpersonal relationship has long been recognized as a significant context contributing to child mental health and development (19). Family functioning theory (20) articulates the way where stress within a family system negatively impacts parental involvement, family cohesion, and family adaptability, and impairs children's internalizing and externalizing psycho-social wellbeing (21). Parent-child communication is emphasized as an important indicator of the family functioning (2), and is also widely considered as a dynamic procedure, where family members can exchange their emotions with joy and confidence. The beneficial effects of parent-child communication on LBC's mental health have been well-established in previous studies (2, 22). Healthy parent-child communication is also associated with better psychological resilience (23) and a higher level of school satisfaction of children (24), and a strong predictive factor of children's self-rated health (25).

In addition to parents' level, the school's interpersonal relationships also play an important role in children's development (26). As noted, teachers' support can reduce LBC's anxiety (27), loneliness (28) and also enhance LBC's psychological resilience and autonomous motivation (29). Peers' support can have a similar effect (30). With friends' companionship, children acquire opportunities for emotional expressions and display lower levels of depressive symptoms (31). Indeed, the care from peers has been found to help children avoid lonely and depressed feelings in contexts of social isolation. Generally speaking, children spend more time at school than at home. Therefore, teachers' and peers' care and supports are important resources for LBC's development and health (29). While the effects of parent-child communication, teacher-child/ peer-child relationships have been well addressed among LBC and MC, little has been done among children with diverse migration experiences.

### The Current Study

To better understand the Chinese rural children's mental health, especially those with diverse migrant experiences, this study hypothesized that: (1) Compared to children without any migrant and left-behind experience, LBC with previous migrant experiences would exhibit more depressive symptoms, poorer self-reported health, and a higher prevalence of suicidal ideation; (2) Good interpersonal relationships can offset the negative effects of migrant experiences on LBC's self-rated health, suicidal ideation and depressive symptoms.

## Methods

### Participants and Procedure

This cross-sectional study was conducted in Anhui and Zhejiang province in China between November to December 2020. Anhui, a relatively underdeveloped southeastern province, has 16 million migrant workers and 4.5 million LBC (2); Zhejiang is a developed coastal province but its western mountainous area is relatively underdeveloped and has numerous LBC. We purposely chose one underdeveloped county in each province (Kaihua in Zhejiang, Nanling in Anhui) and randomly selected three towns from each county, and then selected two schools (one primary school and one junior high school) from each town randomly. In each school, all classes in the targeted grades (grade 5–6 in primary school and grade 7–8 in junior high school) were selected. All students in the selected classes were invited to participate. Across the twelve schools, 2,931 out of 3,025 eligible students completed the questionnaire, representing a response rate of 96.9%. We discarded 122 questionnaires due to the lack of information about parental migration trajectories and migrant experience about him/herself. We then excluded 486 children from incomplete families (e.g., parents have divorced, passed away, etc.). The final sample of the present study was consisted of 2,323 participants.

Ethical approval for this study was obtained from the Ethics Committee of the School of Public Health at Zhejiang University (NO. ZGL201804-2) and the consent and permission were obtained from the local government and presidents of the sample schools. Mandarin was used in the survey to collect data as precise (reliable) as possible. Before the survey, an informed consent form enclosing a detailed description of this study was provided to both the eligible students and their guardians. On the day of the survey, two research assistants were present and conducted the survey during the lunch break in classrooms. In the beginning, the contents of the survey were introduced by research assistants. Participants were informed that their participation was confidential and anonymous, that they could quit at any time even after consent was obtained. Then, one research assistant gave instructions for filling the questionnaire and the other maintained the classroom order. Participants answered the questions by themselves and were informed that they were not allowed to discuss with each other. Considering the that the survey covered the student's privacy information, the questionnaire were completed by children without teachers' presence during the whole process to ensure the true and distraction-free responses. Anonymity and confidentiality were assured. The questionnaire took the participants about 30 minutes (32).

### Measures

#### Socio-Demographic Variables

Socio-demographic information included: province, age, sex, grade, household wealth level, and parental highest education level. Household wealth level was defined as the economic level of the family and then was coded as “top/upper-middle,” “middle” and “lower-middle/low.” Parental highest education level referred to the higher education level of their two parents and was characterized as “primary school or below,” “middle school,” and “high school or above.”

#### Previous Migration Experience and Current Left-Behind Status

Previous migration experience and current left-behind status were identified by asking the following two questions: “Since you were born, have you ever studied or lived in the place where your parent (s) work for at least 6 months, except winter or summer vacation or other holidays?” and “Has your parent (s) migrated into other places for work and been absent for over 6 months?” Children were classified as ME-LBC if they respond “Yes.” to both questions. Children were classified as ME-NLBC if they respond “Yes.” to the first question and “No.” to the second question. Children were classified as LBC if they respond “No.” to the first question and “Yes.” to the second question. Children were classified as NLBC if they respond “No.” to both questions.

#### Interpersonal Relationships

##### Peer's Level

According to the recent study conducted in China (33), the peer's relationship was measured by the following question: “In general, how much do you feel your friend's care about you?” The response options were “Very much / affirmative,” “Neutral” and “Seldom/none.”

##### Teacher's Level

Children were asked by the following question: “In general, how much do you feel your teacher's care about you?” In the analyses, responses were divided into three types, similar to the peer's level.

##### Parents' Level

The communication between parent and child was assessed by the Chinese version of the Parent-Adolescent Communication Scale (PACS) (34, 35). This 20-item scale is composed of two sub-scales: one that measures the communication openness with parents, and the other that assesses the degree of communication problem with parents. Each sub-scale has 10 items. Each item is scored on a 5-point Likert scale from 1 (strongly disagree) to 5 (strongly agree). A higher score of the openness and a lower score of the problem indicate better parent-adolescent communication. In the current study, the Cronbach's alpha of father-adolescent and mother-adolescent communication scales was 0.903 and 0.919, respectively.

#### Health Outcomes

##### Self-Rated Health (SRH)

SRH was measured by one question from the SF-36. The SF-36 questionnaire is a brief self-administered questionnaire, which is widely used by many countries in the world, and also translated and validated to Chinese version (36). Children were asked to assess their general health with the question “How do you rate your current health status?” Response options were “excellent,” “very good,” “good,” “fair” or “poor” (37). Responses with “good,” “fair” and “poor” were coded 0 and “excellent” and “very good” were coded 1 in the current study.

##### Depression

The Chinese version of the Children's Depression Inventory (CDI) was used to measure depressive symptoms of children in the present study. The CDI is one of the most widely used instruments in gauging children's depressive symptoms across the world (38). It is a self-rated symptom-oriented 27-item scale and each item is scored on a three-point Likert scale. Depression was analyzed as both continuous and categorical variables. The CDI score is used as continuous variable with the mean taken as a measure of depressive tendency in rural children, the total score ranges from 0 to 54, with higher scores corresponding to more severe depressive symptoms (39). While the categorical variable quantifies the prevalence of depression, a score of or above 19 is identified as depression (38, 40). The Chinese version of CDI demonstrates high internal consistency and construct validity in the Chinese context (41), and the Cronbach's alpha in the present study was 0.894.

##### Suicidal Ideation

The Beck Depression Inventory (BDI) is one of the most widely used self-report instruments for the assessment of depression. Children's suicidal ideation was assessed by one item selected from the BDI: “Which of the following options best fits your real situation during the past year?” Previous studies showed that using a single suicide item derived from the Beck's depression scale might be a valid approach to assess suicidal ideation (42). Of the four options, the following three options were identified as a “Yes.” for suicidal ideation: “I had thoughts of killing myself, but I wouldn't carry them out.”, “I had thoughts of killing myself.” and “ I will kill myself when I get a chance.”

### Data Analysis

Firstly, chi-square tests and one-way analysis of variance (ANOVA) were conducted to examine differences of demographic characteristics, interpersonal relationships and health outcomes among four different groups of children. Secondly, binary logistic regressions and linear regressions were performed to examine the association between groups of children and health outcomes (self-rated health, suicidal ideation, and depression), and the association between interpersonal relationships and health outcomes after controlling for the province, age, sex, grade, household wealth level, and parental highest education level. All statistical analyses were performed using SPSS version 20.0 and assumed a statistical significance level of *p* < 0.05.

## Results

The present study included 2,323 children, including 336 ME-LBC, 283 ME-NLBC, 561 LBC, and 1,143 NLBC. Table 1 presents the socio-demographic characteristics of children stratified by groups. Overall, there was no statistically significant difference in the province, age, grade, household wealth level, and parental highest education level among four groups of children. ME-LBC has the highest proportion of boys, followed by LBC, ME-NLBC, and ME-LBC.

**Table 1:** Socio-demographic characteristics of children stratified by groups.

	**ME-LBC^a^ *N* (%)**	**ME-NLBC^b^** ***N* (%)**	**LBC^c^ *N* (%)**	**NLBC^d^** ***N* (%)**	**F or χ2**	***p***
**Province**					0.941	0.816
Anhui	164 (48.8)	142 (50.2)	272 (48.5)	580 (50.7)		
Zhejiang	172 (51.2)	141 (49.8)	289 (51.5)	563 (49.3)		
**Age (Mean, SD)**	11.96 (1.20)	11.91 (1.17)	11.90 (1.20)	11.90 (1.21)	0.247	0.863
**Sex**					7.943	0.047
Male	181 (55.0)	150 (54.9)	286 (52.2)	539 (48.1)		
Female	148 (45.0)	123 (45.1)	262 (47.8)	582 (51.9)		
**Grade**					1.522	0.677
Grade 5 Grade 6	153 (45.7)	132 (46.6)	278 (49.6)	546 (47.8)		
Grade 7 Grade 8	182 (54.3)	151 (53.4)	282 (50.4)	597 (52.2)		
**Household wealth level**					9.861	0.131
Top/upper-middle	84 (25.1)	74 (26.2)	125 (22.3)	320 (28.0)		
Middle	221 (66.2)	183 (64.9)	386 (68.9)	748 (65.6)		
Lower-middle/ low	29 (8.7)	25 (8.9)	49 (8.8)	73 (6.4)		
**Parental highest education level**					9.349	0.155
Primary school or below	36 (10.8)	34 (12.1)	55 (9.9)	116 (10.2)		
Middle school	197 (59.2)	158 (56.0)	354 (63.6)	647 (56.9)		
High school or above	100 (30.0)	90 (31.9)	148 (26.6)	375 (33.0)		

a *Left-behind children with previous migration experiences.*

b *Non-left-behind children with previous migration experiences.*

c *Left-behind children.*

d *Non-left-behind children*.

Table 2 shows the interpersonal relationships of children stratified by groups. There were significant differences between different children's groups with respect to care from friends (*p* = 0.003), care from teachers (*p* = 0.009), and problem subscale (*p* = 0.006) of mother-adolescent communication. ME-NLBC reported significantly higher scores in mother-adolescent communication problem than LBC (27.31 ± 7.91 vs. 26.13 ± 7.80), and also than NLBC (27.31 ± 7.91 vs. 25.50 ± 7.70).

**Table 2:** Interpersonal relationships of children stratified by groups.

	**ME-LBC^a^ (1)**	**ME-NLBC^b^ (2)**	**LBC^c^ (3)**	**NLBC^d^ (4)**	**F or χ2**	***p***	**PC^&^**
**Care from friends** *N* (%)					20.146	0.003	(3,4)
Very much/affirmative	242 (72.0)	208 (73.8)	372 (66.5)	863 (75.6)			
Neutral	83 (24.7)	63 (22.3)	168 (30.1)	259 (22.7)			
Seldom/none	11 (3.3)	11 (3.9)	19 (3.4)	20 (1.8)			
**Care from teachers N (%)**					17.057	0.009	(3,4)
Very much/affirmative	246 (73.7)	212 (76.0)	407 (73.1)	895 (78.7)			
Neutral	82 (24.6)	56 (20.1)	142 (25.5)	225 (19.8)			
Seldom/none	6 (1.8)	11 (3.9)	8 (1.4)	17 (1.5)			
**Mother-adolescent communication (Mean, SD)**					
Openness subscale	35.48 (9.94)	35.04 (9.82)	35.33 (9.87)	36.02 (9.77)	1.126	0.337	
Problem subscale	26.10 (7.78)	27.31 (7.91)	26.13 (7.80)	25.50 (7.70)	4.200	0.006	(2,3) (2,4)
**Father-adolescent communication (Mean, SD)**					
Openness subscale	36.12 (10.08)	35.42 (10.59)	36.14 (10.01)	36.22 (10.04)	0.482	0.695	
Problem subscale	24.62 (8.18)	24.91 (8.13)	23.91 (7.93)	24.26 (8.00)	1.158	0.324	

a *Left-behind children with previous migration experiences.*

b *Non-left-behind children with previous migration experiences.*

c *Left-behind children.*

d *Non-left-behind children.*

& *PC indicates the significance of pairwise comparisons in the post hoc analysis*.

Table 3 shows the health outcomes of children stratified by groups. ME-LBC reported a significantly lower prevalence of being excellent/ very good in self-rated health than NLBC (63.0 vs. 70.8%). ME-LBC reported a significantly higher prevalence of depression than NLBC (28.9 vs. 21.2%). ME-LBC reported significantly higher scores of depression than NLBC (14.42 ± 8.51 vs.12.71 ± 8.22).

**Table 3:** Health outcomes of children stratified by groups.

	**ME-LBC^a^ (1)**	**ME-NLBC^b^ (2)**	**LBC^c^ (3)**	**NLBC^d^ (4)**	**F or χ2**	***p***	**PC^&^**
**Self-rated Health** ***N*** **(%)**					10.593	0.014	(1, 4)
Excellent/very good	211 (63.0)	197 (69.6)	365 (65.1)	808 (70.8)			
good/fair/poor	124 (37.0)	86 (30.4)	196 (34.9)	333 (29.2)			
**Suicidal intention** ***N*** **(%)**					2.241	0.524	
Yes	94 (28.1)	87 (31.0)	164 (29.3)	308 (27.0)			
No	241 (71.9)	194 (69.0)	396 (70.7)	834 (73.0)			
**Depression (Categorical)** ***N*** **(%)**				10.302	0.016	(1, 4)
Yes	85 (28.9)	56 (22.5)	135 (26.5)	216 (21.2)			
No	209 (71.1)	193 (77.5)	374 (73.5)	802 (78.8)			
**Depression (Continuous) (Mean, SD)**						
CDI score	14.42 (8.51)	13.92 (8.26)	13.70 (8.43)	12.71 (8.22)	4.308	0.005	(1, 4)

a *Left-behind children with previous migration experiences.*

b *Non-left-behind children with previous migration experiences.*

c *Left-behind children.*

d *Non-left-behind children.*

& *PC indicates the significance of pairwise comparisons in the post hoc analysis*.

Tables 4, 5 show the regression analysis results for health outcomes, interpersonal relationships, and groups with adjustments for the province, age, sex, grade, household wealth level, and parental highest education level. In Table 4, compared with NLBC, ME-LBC (OR = 0.69, 95% CI [0.53, 0.91], *p* < 0.01) reported significantly poorer self-rated health. In Table 5, when adjustments for interpersonal relationships were introduced, ME-LBC (OR = 0.72, 95% CI [0.53, 0.97], *p* < 0.05) reported significantly poorer self-rated health.

**Table 4:** Regression coefficients for health outcomes and groups with adjustments for social-demographic characteristics.

	**Self-rated health**	**Suicidal intention**	**Depression (Categorical)**	**Depression (Continuous)**
	**OR (95%CI)**	**OR (95%CI)**	**OR (95%CI)**	**β (95%CI)**
**Group (Ref: NLBC^d^)**			
ME-LBC^a^	0.69 (0.53, 0.91)^**^	1.09 (0.82, 1.45)	1.44 (1.06, 1.96)^*^	1.49 (0.42, 2.55)^*^
ME-NLBC^b^	0.92 (0.68, 1.25)	1.31 (0.97, 1.77)	1.01 (0.71, 1.44)	1.08 (−0.06, 2.21)
LBC^c^	0.77 (0.62, 0.97)^*^	1.15 (0.91, 1.46)	1.40 (1.08, 1.80)^*^	0.95 (0.08, 1.82)^**^

*With adjustments for province, age, sex, grade, household wealth level, and parental highest education level.*

a *Left-behind children with previous migration experiences.*

b *Non-left-behind children with previous migration experiences.*

c *Left-behind children.*

d *Non-left-behind children.*

* *p < 0.05,*

** *p < 0.01*.

**Table 5:** Regression coefficients for health outcomes, interpersonal relationships and groups with adjustments for social-demographic characteristics.

	**Self-rated health**	**Suicidal intention**	**Depression (Categorical)**	**Depression (Continuous)**
	**OR (95%CI)**	**OR (95%CI)**	**OR (95%CI)**	**β (95%CI)**
**Group (Ref: NLBC^d^)**				
ME-LBC^a^	0.72 (0.53, 0.97)^*^	1.05 (0.76, 1.46)	1.26 (0.86, 1.84)	0.90 (0.02, 1.77)^*^
ME-NLBC^b^	0.96 (0.70, 1.33)	1.25 (0.90, 1.75)	0.83 (0.55, 1.25)	0.10 (−0.83, 1.03)
LBC^c^	0.85 (0.66, 1.08)	1.10 (0.84, 1.44)	1.25 (0.92, 1.70)	0.20 (−0.52, 0.91)
**Care from friends (Ref: Seldom/ none)**				
Very much/affirmative	1.97 (1.07, 3.64)^*^	0.74 (0.39, 1.42)	0.14 (0.06, 0.31)^***^	−7.19 (−9.12, −5.25)^***^
Neutral	1.12 (0.60, 2.07)	0.80 (0.41, 1.54)	0.25 (0.11, 0.58)^*^	−4.37 (−6.32, −2.41)^***^
**Care from teachers (Ref: Seldom/ none)**				
Very much/affirmative	1.02 (0.49, 2.14)	0.35 (0.16, 0.77)^**^	0.35 (0.15, 0.82)^*^	−4.85 (−7.06, −2.64)^***^
Neutral	0.74 (0.35, 1.55)	0.48 (0.22, 1.06)	0.60 (0.25, 1.44)	−3.60 (−5.84, −1.37)^**^
**Mother-adolescent communication**				
Openness subscale	1.01 (1.00, 1.03)	0.98 (0.96, 0.99)	0.95 (0.94, 0.97)^***^	−0.17 (−0.21, −0.12)^***^
Problem subscale	0.96 (0.94, 0.98)^***^	1.08 (1.06, 1.11)^**^	1.09 (1.06, 1.11)^***^	0.28 (0.23, 0.34)^***^
**Father-adolescent communication**				
Openness subscale	1.03 (1.01, 1.04)^***^	0.97 (0.96, 0.99)^***^	0.97 (0.96, 0.99)^***^	−0.11 (−0.15, −0.07)^***^
Problem subscale	1.02 (1.00, 1.04)^*^	0.99 (0.98, 1.01)	1.01 (0.99, 1.03)	0.04 (−0.01, 0.09)

*With adjustments for province, age, sex, grade, household wealth level, and parental highest education level.*

a *Left-behind children with previous migration experiences.*

b *Non-left-behind children with previous migration experiences.*

c *left-behind children.*

d *Non-left-behind children.*

* *p < 0.05,*

** *p < 0.01,*

*** *p < 0.001*.

In Table 4, compared with NLBC, ME-LBC (OR = 1.44, 95% CI [1.06, 1.96], *p* < 0.01) reported significantly higher prevalence of depression. In Table 5, when adjustments for interpersonal relationships were introduced, results were no longer significant.

In Table 4, compared with NLBC, ME-LBC (β = 1.49, 95% CI [0.42, 2.55], *p* < 0.05) reported significantly higher scores of depression. In Table 5, when adjustments for interpersonal relationships were introduced, ME-LBC (β = 0.90, 95% CI [0.02, 1.77], *p* < 0.05) reported significantly higher scores and the difference between NLBC and LBC was no longer significant.

Table 5 also presents the association between interpersonal relationship with children's health outcome. In family level, the mother-adolescent communication's problem was associated with worse self-rated health, higher level of suicidal intention and depression. In contrast, the openness of father-adolescent communication associated with better health status. In school level, the better peer and teacher's relationship was associated with lower level of depression among all the groups of children.

## Discussion

To the best of our knowledge, there was very limited evidence that focused on the health status of Chinese rural children with both migrant and left-behind experiences. Based on a sample collected from two Chinese provinces with widely differing socioeconomic development levels, our original hypotheses were partially confirmed. Firstly, left-behind children with previous migrant experiences exhibited poorer self-rated health and more depression compared with children without any left-behind or migrant experiences. Secondly, better interpersonal relationships including peers' supports, teachers' supports, and parent-adolescent communication can partially offset the negative effects of previous migrant experiences on ME-LBC's self-rated health and fully offset the negative effects of left-behind status on LBC's self-rated health. Thirdly, better interpersonal relationships including peers' supports, teachers' supports, and parent-adolescent communication were associated with better self-rated health, less depressive symptoms and suicidal intention.

Children with mixed experiences of being migrant and left-behind were vulnerable. One study conducted in Guizhou province with 701 participants (43) also indicated that children with mixed experiences of being previously migrant and being presently left-behind might had poor mental health than ones with only being left-behind or being migrant. Using Bourdieu's Field Theory as a guiding framework, the social capital that migrant families accumulate while working in cities can not easily be transferred to the context of rural places. When migrant children are “forced” to return to their hometowns, they carry a different set of dispositions (habitus) into the rural education system (field) (17) including different curriculum content, teaching practices, and performance standards (17). Thus, ME-LBC can face huge challenges in adapting to rural life without parents' presence. Previous studies demonstrated that ME-NLBC adapted better to their new schools and communities in rural areas than ME-LBC did (16, 17). In this regard, LBC with migration experiences might be vulnerable (43).

Furman and Buhrmester's (44) social provisional theory highlights that interpersonal relationships (i.e., parental relationship and friendship) are important determinants of the wellbeing and development of children. Positive family communication has been identified as an important protective factor in the mental health and wellbeing of young people (45). One study conducted in Chongqing province (China) showed that a lower frequency of parent–child communication was a risk factor for depression symptoms among LBC (22) In addition to the family environment, the relationship with friends also constitutes an important developmental context for children. Given the huge importance of friend companionship in children's lives, the research by Zhao et al. has shown the significance of friends' support in decreasing the LBC's depression and loneliness in rural China, confirming the validity of the associations between friendship quality and children's emotional outcomes (22). Teacher support can also reduce LBC's anxiety levels in the school setting (27).

Self-rated Health is considered as a subjective indicator that is used to reflect the result of general health (46). Previous studies have shown that SRH is a stable health outcome measured from adolescence to early adulthood (47, 48). Correspondingly, the SRH tool has been applied to LBC in Ghana and Nigeria (49). Our results also suggested that, in general, the ME-LBC have persistently worse self-reported health than NLBC. During the process of adaptation to new situations, ME-LBC who migrated from urban to rural areas are more likely to face challenges from the school setting and the family structure different from their residence before (46).

Given the significant effects of parental-child communication on children's health status, intervention and training programs can be provided for parents and guardians to enhance their skills in caregiving and communicating with children as a way to foster the growth of the family function. It is important to help children maintain positive and regular communication with their migrant parents and present caregivers. Next, considering the significant effects of peers' care and companionship on children's mental health, special attention must be paid to help children with migration experiences have more connections with their friends and classmates in the school. It is important to help them acquire social skills for reciprocal friendships to gain more social support. It is also important to provide training programs for teachers so that they can provide more care and spend more time on ME-LBC, not only on the academic performance but also on their emotional care and support. Schools may be the suitable settings for mental health programs in rural areas with ever more unpredictable home environments (50).

Several limitations of this study should be noted. Firstly, the present study was student self-administrated which may lead to underestimates of the suicidal ideation incidence and depression incidence out of social desirability. Future studies might better collect data from multiple informants (e.g., parents or caregivers) to achieve a more sophisticated data set (51). For example, to understand the situation of suicidal ideation of rural children, more factors should be taken into consideration, including domestic violence and parents' history of mental illness. Secondly, the generalizability of our results can be limited since we only included students from two provinces in China. Thirdly, we did not include the return time or migration time point and duration of migration and return, nor did we include details of parental absence in our studies, which might further enrich the present study.

Despite these potential limitations, our findings have some important contributions to the current research literature. Firstly, the present study expands the knowledge that Chinese rural children are not a homogeneous population (43). And we found that ME-LBC were the more depressed group. It is necessary to distinguish the diverse migration status and to explore the differentiated protective mechanisms of their health status, especially of the current LBC who had previous migration experiences. As mentioned above, this group of children was described as “a doubly disadvantaged group” due to the separation from parents if they returned alone and also the need for re-adoption to new community lifestyles and rebuilding the interpersonal connection (17). Second, the government needs to create greater opportunities for children to unite with their parents in urban areas. Third, considering that LBC, especially those LBC with previous migrant experiences, are the most vulnerable to emotional difficulties, special attention must be paid to these children to help them overcome the challenges induced by the parental absence and re-adaptation to the new environment.

Finally, the issue of health status and wellbeing of rural children might receive more extensive attention from the whole society. At the international level, the United Nations *Transforming our world the 2030 agenda for sustainable development* (14) through its sustainable development goals (particularly SDG 17) encourage all the countries to implement research practice and policies to assure the rights, health and wellbeing of migrant workers and their children. At the national level, China has initiated key programs in recent years aiming at providing better health services to the LBC. The National Mental Health Work Plan (2015–2020) aims to establish psychological counseling rooms in all schools and to increase awareness of psychological wellbeing (14). In 2019, the “Child Welfare Officer” project was promoted nationwide by the Ministry of Civil Affairs of the People's Republic of China (52) in order to improve the better growth and development of children, especially vulnerable Chinese rural children. It is important that we need to face the heterogeneity of rural children and their health status. Given growing evidence of the influence of sex and gender on children's mental health (53, 54), it will be important in future studies of Chinese rural children's mental health to consider both sex and gender-related influences. Seeking effective intervention strategies also should be considered by the government, society, and social welfare organizations. More studies should verify the effectiveness of evidence-based intervention's effect in the future.

## Conclusion

The present study showed that previous migration experiences significantly increase the risk of depression and poorer self-rated health among rural children in China. And the good interpersonal relationships was associated with a lower level of depression and better self-rated health. Interventions aiming at improving relationships of parents, peers and teacher can exert great benefits on left-behind children with previous migration experiences.

## Data Availability Statement

The raw data supporting the conclusions of this article will be made available by the authors, without undue reservation.

## Ethics Statement

The studies involving human participants were reviewed and approved by the Ethics Committee of the School of Public Health at Zhejiang University (China). Written informed consent to participate in this study was provided by the participants' legal guardian/next of kin.

## Author Contributions

FW conceptualized the study conception and design. FW, GZ, MW, JX, JL, and HA collected the data. GZ and FW conceived data interpretation and performed all statistical analyses. GZ drafted manuscript while MW, JX, JL, and HA critically revised the manuscript for important substantial revisions. All authors gave their final approval and agree to be responsible for all aspects of the work. All authors contributed to the article and approved the submitted version.

## Funding

This research was supported by the Zhejiang University Zijin Talent Project, the China Postdoctoral Science Foundation (Grant Number 2020M671777) and the National Social Science Fund of China (Grant Number 21CRK015). The funding institution had no role in the study design, the collection, analysis and interpretation of the data, and the decision to publish or preparation of the manuscript.

## Conflict of Interest

The authors declare that the research was conducted in the absence of any commercial or financial relationships that could be construed as a potential conflict of interest.

## Publisher's Note

All claims expressed in this article are solely those of the authors and do not necessarily represent those of their affiliated organizations, or those of the publisher, the editors and the reviewers. Any product that may be evaluated in this article, or claim that may be made by its manufacturer, is not guaranteed or endorsed by the publisher.
